# Preexisting Comorbidities Predicting COVID-19 and Mortality in the UK Biobank Community Cohort

**DOI:** 10.1093/geroni/igab046.1329

**Published:** 2021-12-17

**Authors:** Janice Atkins, Jane Masoli, Joao Delgado, Luke Pilling, Chia-Ling Kuo, George Kuchel, David Melzer

**Affiliations:** 1 University of Exeter Medical School, University of Exeter, England, United Kingdom; 2 University of Exeter, Exeter, England, United Kingdom; 3 University of Exeter Medical School, Exeter, England, United Kingdom; 4 University of Connecticut Health, Farmington, Connecticut, United States

## Abstract

Hospitalized COVID-19 patients tend to be older and frequently have hypertension, diabetes or CHD, but whether these co-morbidities are more common than in the general older population is unclear. We estimated associations between pre-existing diagnoses and hospitalized COVID-19 alone or with mortality (during the first COVID-19 outbreak, tests performed between March 16 and April 26, 2020). In 269,070 UK Biobank participants aged 65+, 507 (0.2%) became COVID-19 hospital inpatients, of which 141 (27.8%) died. Common preexisting co-morbidities in hospitalized inpatients were hypertension (59.6%), history of falls/fragility fractures (29.4%), CHD (21.5%), T2 diabetes (19. 9%) and asthma (17.6%). However, in adjusted models, pre-existing diagnoses of dementia, T2 diabetes, COPD, pneumonia, depression, atrial fibrillation and hypertension emerged as independent risk factors for COVID-19 hospitalization, the first five remaining statistically significant for related mortality. There are specific high risk pre-existing co-morbidities for COVID-19 hospitalization and deaths in community based older men and women.

